# Effects of Dietary Protein Levels on *Macrobrachium rosenbergii* Reared Under a High-Density Culture Model: Growth, Immune Response, Hepatopancreatic Health, and Antistress Capacity

**DOI:** 10.1155/anu/8880975

**Published:** 2025-09-12

**Authors:** Yu Fu, Enhui Chang, Dongxu Luo, Xin Zhang, Haoyue Guo, Anran Wang, Shuyan Miao

**Affiliations:** College of Animal Science and Technology, Yangzhou University, 48 Wenhui East Road, Yangzhou, China

**Keywords:** anti-stress capacity, dietary protein level, growth performance, hepatopancreatic health, high-density culture model, immune response, *Macrobrachium rosenbergii*

## Abstract

Study of precision nutrition provides essential and accurate information on the nutrient requirements for animal growth under various farming modes, to offer guidance for the efficient utilization of compound feed. To evaluate the effects of dietary protein levels on the growth performance, immune response, and health of *Macrobrachium rosenbergii* reared under high-density conditions, prawns in five groups were cultured with a density at 70 prawns/m^3^, and fed diets with varying protein levels (40%, 42.5%, 45%, 47.5%, and 50%) and designated as CP40, CP42.5, CP45, CP47.5, and CP50, respectively. Our findings revealed that the prawns in the CP42.5 and CP45 groups exhibited significantly higher weight gain rates and specific growth rates, whereas the feed conversion ratio (FCR) was significantly lower in these groups (*p* < 0.05). The hepatosomatic index (HSI) of prawns in the CP45 group was significantly higher than the other treatments (*p* < 0.05). The *trypsin* activity in the CP45 group was the highest (*p* < 0.05). Additionally, prawns fed 42.5%–45% protein levels exhibited stronger antioxidant capacity (AOC), with higher total antioxidant capacity (T-AOC), and the activities of catalase (CAT), total superoxide dismutase (T-SOD), and glutathione S-transferase (GST) (*p* < 0.05), along with a substantial reduction in protein carbonyl (PC) levels (*p* < 0.05). Analysis of the expression of apoptosis-related genes and hematoxylin-eosin staining revealed that both insufficient and excessive dietary protein levels significantly led to autophagy in the hepatopancreas. The environmental stress tests demonstrated that the survival rate (SR) of prawns in the CP45 group was significantly higher compared to the other treatments (*p* < 0.05). From a growth and health perspective, our findings revealed that a 42.5%–45% protein level is appropriate for *M. rosenbergii* cultured under high-density (70 prawns/m^3^) conditions.

## 1. Introduction

The commercially cultivated giant freshwater prawn (*Macrobrachium rosenbergii*) holds significant global economic importance [1]. Since, its introduction to China in the 1970s, remarkedly advancements have been made in the breeding and production of *M. rosenbergii*, known for its rapid growth, short cultivation cycle, strong disease resistance, and high-quality meat [2, 3]. The production of *M. rosenbergii* in China has demonstrated a continuous upward trend over the years, with particularly notable development in large-scale industrialized aquaculture systems. One of the key regions for *M. rosenbergii* farming is Jiangsu province, where large-scale, high-density farming systems have been established to meet growing market needs [4]. In addition to the well-established practices of disease prevention and water quality management [5, 6], further efforts are necessary to assess and refine the precise nutritional needs of *M. rosenbergii* under contemporary farming conditions. Protein, as a key nutritional component in aquafeed, plays an essential role in the growth and overall health of animals [7]. Insufficient protein supply can lead to malnutrition and slower growth, affecting production efficiency. Conversely, an excessive protein intake, while potentially promoting growth in the short term, can lead to resource inefficiencies and environmental pollution. The surplus nitrogen and phosphorus from unutilized protein are released into the surrounding water, contributing to eutrophication, which can negatively impact water quality and the sustainability of long-term aquaculture systems [8]. These challenges highlight the need to accurately assess the protein requirements of prawns according to the culture conditions.

The optimal dietary protein level for aquatic animals varies not only by species, but also, according to environmental and management factors, such as temperature [9], salinity [10], stocking density [8], and protein sources [11], all of which can significantly influence growth, health, and feed efficiency in aquaculture systems. For example, a previous study reported that the optimum dietary protein requirement of *M. rosenbergii* reared at a density of 1 juvenile per m^2^ in a total pond area of 200 m^2^ was 32% [12]. However, in most culture areas of Jiangsu, the culture density of *M. rosenbergii* juveniles is typically 120–180 prawns/m^2^, and the water deep often keeps about 1 m. According to the investigation, and based on a 50% survival rate (SR), the density during farming period can reach about 60–90 prawns/m^2^, and the protein content of commercial feed often exceeds 45%–48%, which places significant pressure on the cost of compound feed and the supply of protein sources. In the context of intensive aquaculture systems, especially under high stocking densities, the dietary protein content plays a critical role in ensuring optimal growth, while avoiding excessive nitrogenous waste, which can harm water quality. This underscores the importance of understanding the relationship between stocking density, protein requirements, and feed cost efficiency in order to sustain the profitability of *M. rosenbergii* farming. Hence, there is an increasing need to optimize protein levels to improve feed efficiency, minimize waste, and ensure better economic returns from farming practices.

According to the current farming model and in order to obtain the best production efficiency, this study thereby, evaluated the growth performance, antioxidative capability, and stress response of *M. rosenbergii* fed with different dietary protein levels reared under high-density conditions (70 prawns/m^3^ was suggested). While previous research has established basic protein requirements for *M. rosenbergii*, few studies have examined how varying protein levels interact with high stocking densities to affect growth, stress resilience, and overall health. Ultimately, this research provides a theoretical basis for optimizing feed formulation in *M. rosenbergii* farming, it offers valuable guidance on improving the economic viability of farming in intensive aquaculture systems, helping to enhance feed efficiency, reduce costs, and support the sustainable development of the aquaculture industry.

## 2. Materials and Methods

### 2.1. Diets

In the formulation of the experimental diets, fish meal, shrimp meal, chicken meal, soybean meal, and peanut meal served as the main protein sources, contributing to the overall nutritional profile, while soybean oil and fish oil were incorporated as the primary lipid sources to meet the energy requirements of the prawns. The ingredients and composition of the experimental diets are summarized in Table 1. Five experimental diets with different protein levels (40%, 42.5%, 45%, 47.5%, and 50%) were designed and labeled as CP40, CP42.5, CP45, CP47.5, and CP50 groups, respectively. The diets were prepared as described by Miao et al. [13]. All diets were air-dried at 50°C for 24 h to remove excess moisture and prevent microbial growth, after which they were stored at −20°C to maintain their nutritional integrity and prevent degradation.

### 2.2. Experimental Animals and Culturing Conditions

*M. rosenbergii* was purchased from Sufeng Prawn Factory in Yangzhou, Jiangsu Province. After 2 weeks of acclimation, 1080 prawns each weighing 0.76 ± 0.01 g were randomly assigned to five groups, with four replicates per group and 45 prawns per replicate. A nylon mesh cage (0.8 m × 0.8 m × 1.0 m) was used for each replicate. The cages were placed in cement pond. All prawns were fed the experimental diets at 06:30 and 18:30 for 76 days. During the feeding period, any dead prawns were recorded daily, and uneaten food was carefully removed by siphoning to maintain water quality and prevent overfeeding. Approximately 50% of the water was replaced every day to remove nitrogenous waste generated from protein metabolism. Throughout the feeding experiment, the range of water indicators was as follows: temperature 26–31°C, dissolved oxygen > 6 mg/L, pH 7.6–8.2, nitrite nitrogen < 0.1 mg/L, and total ammonia–nitrogen < 0.1 mg/L. Water temperature was measured using an alcohol thermometer and maintained within the desired range primarily through regular water exchanges, which helped regulate temperature fluctuations. Dissolved oxygen was measured with a portable dissolved oxygen meter, and aeration pumps were adjusted to ensure adequate oxygenation, particularly during feeding. The pH levels were monitored twice daily using a handheld pH meter. Nitrite nitrogen and total ammonia–nitrogen levels were assessed regularly using colorimetric test kits and ammonia test kits, respectively, with partial water changes employed to maintain low levels of these compounds.

### 2.3. Sample Collection and Indicator Analysis

Following the feeding trial, the prawns were subjected to a 24 h fasting period. Prior to sampling, all prawns were anesthetized with tricaine methanesulfonate (MS-222; 100 mg/L) to minimize handling stress and ensure animal welfare. After anesthesia, prawns were individually counted, weighed, and measured to evaluate their health status. The data obtained were then used to calculate key performance indicators, including the weight gain rate (WGR), specific growth rate (SGR), and SR.

Hepatopancreas samples were collected from 12 prawns per replicate. Each prawn was anesthetized with MS-222 (100 mg/L) before dissection. Each hepatopancreas was individually weighed to calculate the hepatosomatic index (HSI). After weighing, tissues from three prawns were each divided into two portions: one was fixed in Bouin's solution for histological examination and the other was stored at –80 °C for gene expression analysis. The hepatopancreas samples from the remaining nine prawns were pooled and evenly divided into three portions, which were stored at –80 °C for subsequent analysis of antioxidant enzyme activities and *trypsin* activity.

Hemolymph was collected from the pericardial cavity of prawns using sterile syringes to minimize contamination. The hemolymph was then mixed with an anticoagulant solution at a 1:1 volume ratio to prevent clotting. For each replicate, hemolymph was collected from six prawns and pooled into a single tube as one sample for subsequent analysis. Serum was separated by centrifugation and stored at 4°C. The anticoagulant solution was prepared according to the protocol outlined by Guo et al. [14], ensuring its efficacy in inhibiting clotting.

The activities of *trypsin*, catalase (CAT), total superoxide dismutase (T-SOD), glutathione S-transferase (GST), alanine aminotransferase (ALT), aspartate aminotransferase (AST), and the level of malondialdehyde (MDA) and total antioxidant capacity (T-AOC), and content of protein carbonyl (PC), nitric oxide (NO), total protein (TP), and albumin (ALB) were determined using commercial kits (Nanjing Jiancheng Bioengineering Institute, Nanjing, China) according to the manufacturer's instructions.

Gene-specific primers targeting key genes, such as B-cell lymphoma-2 (*bcl-2*), *caspase2*, *caspase3*, heat shock protein (*hsp*)60, *hsp70*, *hsp90*, and nuclear factor kappa-B (*nf-κB*), were sourced from Tsingke Biological Technology, Beijing, China. Quantitative real-time PCR (qPCR) experiments were conducted following the method described by Miao et al. [15]. Quantitative gene expression analysis was performed using the 2^-ΔΔCt^ method to calculate the changes in expression [16]. The corresponding primer sequences are provided in Table 2.

### 2.4. Ammonia–Nitrogen and High-Temperature Stress Tests

Acute ammonia–nitrogen challenge experiments were conducted immediately after collecting the samples from the feeding trial. Residual prawns from each treatment were collected, focusing on individuals with good motility and intact limbs. A total of 40 prawns from each feeding treatment were randomly selected and redistributed into cylindrical tanks (10 L), with 10 prawns per tank. The ammonia–nitrogen concentration was set according to the 48 h median lethal concentration (LC_50_) determined in previous studies [17] and preliminary experiments. Ammonia–nitrogen levels were carefully maintained using a 10 g/L NH_4_Cl solution to achieve concentrations below 100 mg/L of ionized ammonia–nitrogen, ensuring the conditions simulated acute ammonia toxicity, while avoiding extreme toxicity that might overwhelm the prawns' physiological response. To ensure optimal conditions during the experiment, the prawns were fasted for the entire exposure period. Mortality counts were recorded at 24 and 48 h.

The grouping procedure was consistent with that of the ammonia–nitrogen stress test, with prawns being gradually exposed to increasing temperatures up to 38.5°C. They were then maintained at 38.5°C for 24 h. Prawn health was closely monitored, and mortalities were documented at intervals of 4, 8, 12, 16, 20, and 24 h.

### 2.5. Statistical Analysis

The growth and morphometric indices, including WGR, SGR, feed conversion ratio (FCR), HSI, condition factor (CF), feed intake (FI), and SR, were calculated using the formulas provided.

  $$\begin{array}{c} \text{WGR }\left(\text{\% }\right)=100\times \left(\text{average final weight}-\text{average initial weight}\right)/\text{average initial weight}, \end{array}$$

  $$\begin{array}{c} \text{SGR }\left(\text{\%/d }\right)=100\times \left(\text{Ln}\left(\text{average final weight}\right)-\text{Ln}\left(\text{average initial weight}\right)\right)/\text{experiment days}, \end{array}$$

  $$\begin{array}{c} \text{FCR}=\text{feed consumed}/\left(\text{average final weight}-\text{average initial weight}\right), \end{array}$$

  $$\begin{array}{c} \text{HSI }\left(\%\right)=100\times \text{hepatopancreas weight}/\text{average final weight}, \end{array}$$

  $$\begin{array}{c} \text{CF }\left(g/{cm}^{3}\right)=100\times \text{average final weight}/{\text{average terminal body length}}^{3}, \end{array}$$

  $$\begin{array}{c} \text{FI}\left(g\right)=\text{feed consumed}/\text{final prawns number}/\text{experiment days}, \end{array}$$

  $$\begin{array}{c} \text{SR }\left(\%\right)=100\times \text{final prawns number}/\text{initial prawns number}. \end{array}$$

Data analysis was performed using Microsoft Excel 2021 and SPSS 27.0 (IBM, USA). Descriptive statistics, including mean and standard deviation (SD), were calculated for each treatment group. One-way ANOVA was used to compare group means, followed by Duncan's test for pairwise comparisons to identify significant differences. The results are presented as means ± SD in figures and as standard error of the mean (SEM) in tables, with statistical significance set at *p*  < 0.05. Additionally, a trend analysis was performed using orthogonal polynomial contrasts to assess both linear and quadratic effects, enabling a comprehensive evaluation of the response pattern across varying levels of the experimental factors.

## 3. Results

### 3.1. Growth Performance, Feed Utilization Efficiency, and Hepatopancreas Index of M. rosenbergii

Different dietary protein levels significantly affected the WGR, SGR, FCR, HSI, and CF of *M. rosenbergii* (*p* < 0.05). In the CP42.5 and CP45 groups, the SGR and WGR of *M. rosenbergii* were significantly higher than those in the CP40, CP47.5, and CP50 groups (*p* < 0.05, Table 3). In contrast, the FCR in CP42.5 and CP45 groups was significantly lower than that in other treatments (*p* < 0.05). Additionally, the HSI of the CP45 group was significantly higher than that of other experimental treatments (*p* < 0.05). There were no significant differences in FI and SR among the experimental treatments (*p* > 0.05). Results revealed both linear and quadratic trends observed for WGR, SGR, and FCR.

### 3.2. Activities of the Trypsin

**Figure 1:**
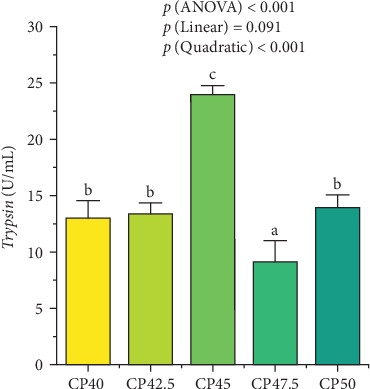
Effects of different protein levels on the activities of digestive enzymes in the hepatopancreas of *M. rosenbergii*. Values are expressed as means ± SD. (*n* = 4). Different letters indicate significant differences (*p* < 0.05). CP40, 40% protein; CP42.5, 42.5% protein; CP45, 45% protein; CP47.5, 47.5% protein; CP50, 50% protein.

The *trypsin* activity in the CP45 group was also the highest among all treatments (*p*  < 0.05). Conversely, prawns in the CP47.5 group exhibited the lowest *trypsin* activity than the other treatments (*p*  < 0.05, Figure 1). Additionally, a quadratic effect of dietary protein levels on *trypsin* activity was found.

### 3.3. Hepatopancreas and Hematologic Antioxidant-Related Parameters

The evidence suggested that the T-AOC level was highest in the CP45, CP47.5, and CP50 groups compared to the other groups. The CAT and T-SOD activities were the highest in group CP45, whereas PC values were the lowest in this treatment (*p* < 0.05, Table 4). The MDA value was the lowest in group CP42.5 compared to the other treatments, whereas group CP47.5 exhibited the highest MDA values. The GST activity in the hepatopancreas was the highest in groups CP45 and CP47.5 compared to the other treatments (*p* < 0.05). Results revealed a quadratic relationship between dietary protein levels and CAT, T-SOD, and PC, whereas both linear and quadratic trends were observed for the correlation between protein levels and GST and T-AOC. A linear trend was observed between protein levels and MDA.

The NO content was the highest in groups CP42.5 and CP45 compared to the other treatments (*p* < 0.05, Table 5). The TP content in the serum was the highest in group CP42.5 compared to the other treatments (*p* < 0.05). Notably, the serum ALT activities were lowest in groups CP42.5 and CP45, whereas the AST activity was the lowest in the CP47.5 compared to the other experimental treatments (*p* < 0.05). The ALB content was the highest in groups CP40, CP42.5, and CP45 compared to the CP47.5 and CP (*p* < 0.05). The results revealed both linear and quadratic trends in the relationship between dietary protein levels and the concentrations of NO, AST, and ALT. Furthermore, a linear relationship was observed between dietary protein levels and the concentrations of TP and ALB.

### 3.4. The Apoptosis-Related Genes

**Figure 2:**
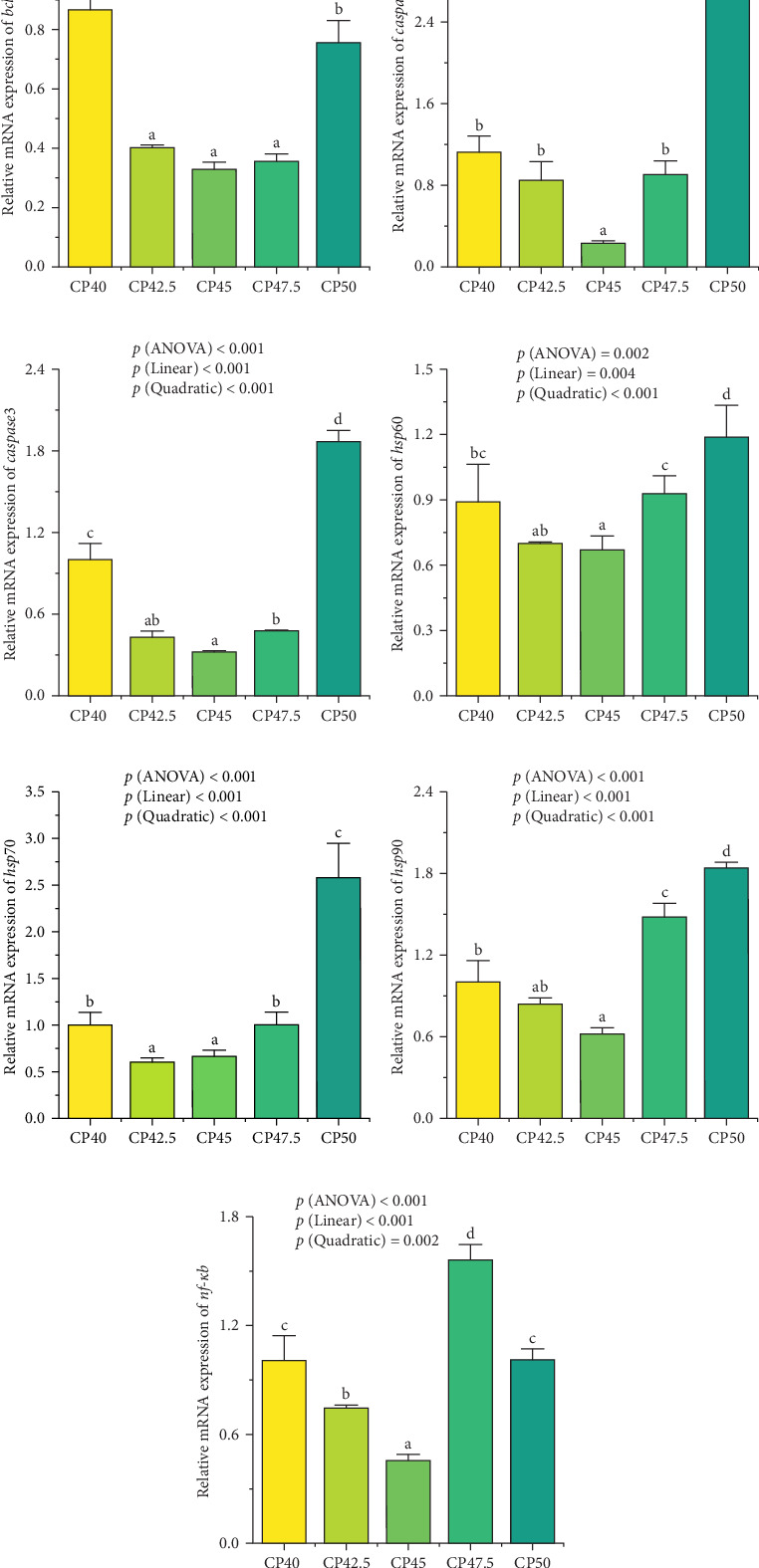
Effects of different protein levels on the expression of apoptosis-related genes in *M. rosenbergii*. (A) *bcl-2*; (B) *caspase2*; (C) *caspase3*; (D) *hsp60*; (E) *hsp70*; (F) *hsp90*; (G) *nf-κb*. Values are expressed as means ± SD. (*n* = 4). Different letters indicate significant differences (*p* < 0.05). CP40, 40% protein; CP42.5, 42.5% protein; CP45, 45% protein; CP47.5, 47.5% protein; CP50, 50% protein.

The expression levels of the gene *bcl-2* in groups CP42.5, CP45, and CP47.5 were significantly lower than those in groups CP40 and CP50 (*p* < 0.05, Figure 2A). Additionally, The relative expression levels of *caspase2*, *caspase3*, *hsp60*, *hsp90*, and *nf-κb* genes in group CP45 were significantly lower than those in remaining groups (*p* < 0.05, Figure 2B-G). The expression levels of *hsp70* in groups CP42.5 and CP45 were significantly lower than those in the remaining groups (*p* < 0.05, Figure 2E). Dietary protein levels exhibited both linear and quadratic trends in the mRNA expression levels of *bcl-2*, *caspase2*, *caspase3*, *hsp60*, *hsp70*, *hsp90*, and *nf-κb*.

### 3.5. Hepatopancreas Histology

**Figure 3:**
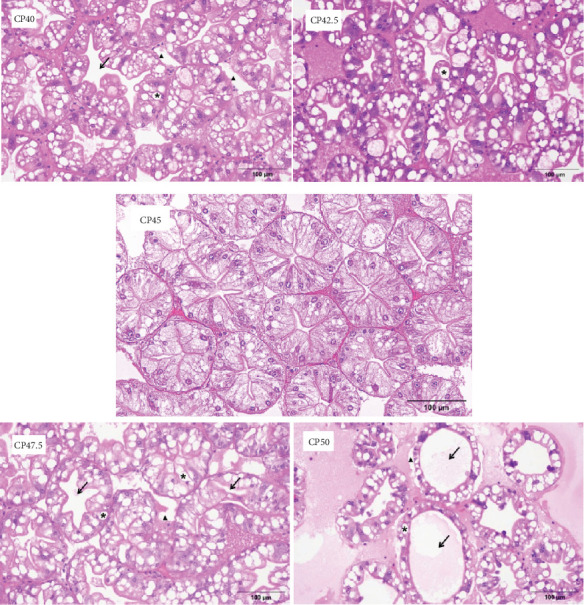
Hematoxylin and eosin staining sections of hepatopancreas. The arrow indicates that the significant deformation of the sinusoidal lumens. The asterisk indicates that vacuoles within the hepatic tubules. The triangle indicates that the fragmented cell membranes. CP40, 40% protein; CP42.5, 42.5% protein; CP45, 45% protein; CP47.5, 47.5% protein; CP50, 50% protein.

Severe damage and pronounced vacuolization of the hepatic tubules were observed in the CP1 group, accompanied by deformation of the sinusoidal lumens and disruption of the intervacuolar connective tissue. Additionally, hepatic tubular rupture and extensive cellular disintegration were evident, with fragmented cell tissue present in the interstices and lumens of the hepatic ducts, resulting in unclear cellular boundaries and impaired tissue organization. In the CP42.5 group, vacuoles persisted within the hepatic tubules; however, the tissue structure remained intact and well-defined. The hepatopancreatic tissue in the CP45 group exhibited an intact tissue structure, with normal sinusoidal luminal morphology, indicating that this protein level supported the optimal tissue integrity. In contrast, at higher dietary protein levels, the CP47.5 group showed a blurring of the basal layer of the hepatic tubules, accompanied by fragmented cell membranes. Examination of the CP50 group revealed significantly expanded and severely damaged hepatic tubules, marked deformation of the sinusoidal lumens, and compromised intervacuolar connective tissue. The boundaries were further obscured, with widened intertubular spaces, loosely arranged cells, and cellular shrinkage (Figure 3).

### 3.6. Survival Curve of Ammonia–Nitrogen Stress and High-Temperature Stress

**Figure 4:**
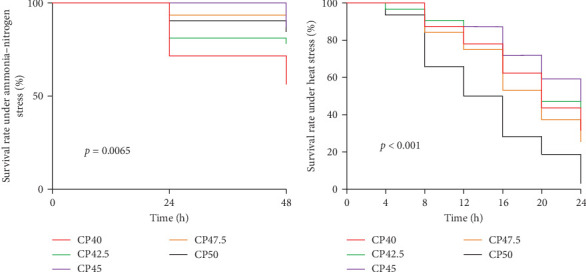
Survival curve of *M. rosenbergii* in response to (A) ammonia–nitrogen stress and (B) high-temperature stress. CP40, 40% protein; CP42.5, 42.5% protein; CP45, 45% protein; CP47.5, 47.5% protein; CP50, 50% protein.

Significant differences in survival rates (SRs) were observed in *M. rosenbergii* under ammonia–nitrogen stress, with the CP45 group showing the highest SR, and the CP40 group the lowest (*p* < 0.05, Figure 4A). Similarly, in the high-temperature stress experiment, the CP45 group had significantly higher SRs than the other groups, while the CP50 group had the lowest (*p* < 0.05, Figure 4B).

## 4. Discussion

Protein serves as a vital nutrient that intricately links physiological structure and function, acting as the foundational substrate for metabolism and biosynthesis across all living organisms [2]. It has been reported that the optimal dietary protein level for *M. rosenbergii* postlarvae falls between 27% and 32% [12]. However, an increase in stocking density has been found to correspond with a higher protein demand in aquatic species, such as blunt snout bream (*Megalobrama ambylcephala*) and indian white shrimp (*Penaeus indicus*) [8, 18]. In high-density aquaculture systems, such as those for *M. rosenbergii* in regions, like Jiangsu Province, China, there has been a significant rise in the demand for high-protein formulated feeds to meet the increased nutritional requirements for growth of prawns under intensive farming conditions. In this study, the growth performance of prawns was significantly influenced by dietary protein levels, with diets containing 42.5% and 45% protein resulting in higher WGR and SGR, suggesting that these protein levels were more effective in promoting growth under the experimental conditions. The results indicate that an adequate protein supply is crucial for the efficient growth of *M. rosenbergii*, particularly in intensive aquaculture systems where protein requirements are elevated due to high stocking densities. Conversely, the feeding of diets with protein levels of 47.5% and 50% negatively impacted growth, which can likely be attributed to excessive protein intake. Additionally, excessive protein levels can also impair digestive efficiency and disrupt metabolic processes, as observed in this study. Excessive protein intake also increases metabolic costs, as the prawns must expend more energy to process and eliminate the surplus protein. This misallocation of energy can hinder optimal growth and, over time, can have negative impacts on feed efficiency and overall health. A similar trend was observed in the activities of *trypsin*. The highest *trypsin* activity was recorded in prawns fed with 42.5% and 45% protein diets, suggesting that these protein levels were utilized more efficiently. In contrast, prawns fed higher protein diets exhibited lower *trypsin* activity, possibly due to digestive inefficiencies associated with excessive protein intake. This finding is consistent with previous studies, which have shown that excessive protein can impair enzyme activity and hinder nutrient absorption, thereby limiting growth [19, 20].

The antioxidative capacity of the hepatopancreas plays a crucial role in maintaining homeostasis in crustaceans [21]. Antioxidative enzymes, such as T-SOD and CAT, are primarily localized in the hepatopancreas and hemocytes, playing critical roles in the antioxidative defense system of aquatic organisms. When tissue peroxidation increases, it is often indicative of a disruption in the organism's intrinsic antioxidative capacity. PC levels are commonly used as markers of oxidative damage to proteins, while MDA levels indicate lipid peroxidation [22]. In this study, the activities of T-SOD and CAT in the hepatopancreas of *M. rosenbergii* initially increased with higher dietary protein levels, but then decreased at higher protein concentrations. Similarly, the levels of MDA and PC first decreased and then increased. These results align with previous studies on species, such as blunt snout bream and common carp (*Cyprinus carpio*) [23]. The initial increase in antioxidative enzyme activity likely reflects an adaptive response to the oxidative stress caused by high dietary protein. However, the subsequent decrease in enzyme activity and the increase in MDA and PC levels at higher protein concentrations suggest that excessive protein may overwhelm the antioxidative defense system, leading to cellular damage. Furthermore, the integrity of cellular membranes was compromised in the high-protein diet groups, as evidenced by the efflux of intracellular fluid. Membrane damage is a clear indicator of deepening cellular injury, and such damage can have serious implications for the overall health and function of cells. The disruption of membrane integrity is often associated with increased cellular permeability, which can lead to the leakage of intracellular contents and ultimately to cell death. These observations support the notion that excessive protein intake can negatively affect the antioxidative capacity of *M. rosenbergii*, leading to increased oxidative stress and impaired cellular function.

Apoptosis, a fundamental and conserved process of programed cell death, is essential for maintaining cellular homeostasis within the hepatopancreas of crustaceans. The regulation of apoptosis is vital for the removal of damaged or dysfunctional cells, thereby ensuring the proper functioning of the organism's physiological systems [24]. *Caspase2* and *caspase3* play pivotal roles in the apoptotic pathway, classified as initiator and executioner caspases, respectively. *Caspase2*, as an initiator caspase, regulates apoptosis through two key pathways: the intrinsic pathway, which is *bcl-2*-regulated, and the extrinsic pathway involving death receptors [25]. *Caspase3*, a downstream executioner *caspase*, plays a critical role in the execution phase by activating the final steps of cell death signaling, leading to the degradation of cellular components and eventual cell death [26]. Both excessive and insufficient dietary protein levels can induce the expression of *caspase2* and *caspase3* in *M. rosenbergii*, leading to apoptosis in hepatopancreatic cells. The expression of *bcl-2*, an upstream regulator in the intrinsic apoptotic pathway, exhibited similar trends to *caspase2* and *caspase3*, further supporting the relationship between dietary protein levels and apoptotic regulation. Heat shock proteins (HSPs) serve pivotal functions in normal physiological conditions and under stress. Under normal physiological conditions, the expression level of HSPs is at a relatively lower level, however, their synthesis within cells can sharply increase under environmental stress, pathology, or physiological stress [27]. A recent study suggested that *hsp60* and *hsp90* interact with *caspase2* and *caspase3*, collectively regulating cellular apoptosis [28]. This study observed a marked upregulation of HSPs, particularly in the high-protein diet groups, which may reflect a stress response triggered by the elevated protein levels. This interaction between HSPs and caspases underscores the dynamic regulatory mechanisms that control apoptosis under stress and nutritional imbalances. The upregulation of *nf-κb* gene expression observed in this study suggests that elevated dietary protein levels may induce an inflammatory response in *M. rosenbergii*. *nf-κb* is a key transcription factor that regulates various immune and inflammatory processes. The increased expression of *nf-κb* could trigger the production of pro-inflammatory cytokines, contributing to tissue damage and further exacerbating oxidative stress, which in turn could promote apoptosis and impair immune function. These findings are consistent with previous research in other fish species, including yellow catfish (*Pelteobagrus fulvidraco*) [29], bighead carp (*Aristichthys nobilis*) [30], and grass carp (*Ctenopharyngodon idella*) [22]. Moreover, the histological examination of hepatopancreas tissue sections corroborated the gene expression results, revealing that the high-protein diet group exhibited more pronounced cellular apoptosis in the hepatopancreas. These histological observations were consistent with the increased expression of apoptosis-related genes, such as *bcl-2*, *caspase2*, and *caspase3*, thus providing direct morphological evidence of the link between high dietary protein levels and enhanced apoptotic activity. Notably, the cellular damage in the high-protein groups involved severe vacuolization of the hepatopancreatic cells, membrane rupture, and fragmentation of cellular structures. Such damage was particularly evident in the CP50 group, where the structural integrity of the hepatopancreas was significantly compromised. This observation not only aligns with the gene expression data but also emphasizes that excessive protein intake can disrupt cellular homeostasis, triggering apoptotic processes. The pronounced apoptosis observed in these groups indicates that an overabundance of protein may surpass the organism's capacity for cellular adaptation, leading to an overload of stress-related signaling pathways and resulting in cellular dysfunction. The observed cellular damage underscores the potential risks of feeding diets with excessively high protein levels, particularly under high-density farming conditions, where the compounded stress from both nutritional and environmental factors may amplify apoptotic responses.

Antistress tests are commonly used to comprehensively assess the health and immune capabilities of animals in response to environmental stressors [31, 32]. In high-density *M. rosenbergii* culture, the use of high-protein feed often leads to elevated ammonia–nitrogen concentrations in the water, which can pose significant challenges to the aquatic environment [33, 34]. Furthermore, during the rapid growth phase in May and June, water temperatures frequently exceed 33–35°C, exacerbating the effects of high stocking densities and high-protein feeding regimes. These elevated temperatures can impose physiological stress on prawns by disrupting their metabolic processes, impairing immune function, and ultimately compromising their growth and SRs. These stressors are common in high-density *M. rosenbergii* culture, especially during the rapid growth phase. To evaluate the combined effects of these environmental stressors, ammonia–nitrogen and high-temperature stress assays were conducted to examine the impact of dietary protein levels on the survival and stress resistance of *M. rosenbergii* under challenging conditions. Our results showed that both excessive and insufficient dietary protein levels significantly impaired prawns' ability to tolerate these environmental stresses, leading to lower SRs. These findings are in line with the results of our immune function and antioxidant capacity (AOC) assays, which further supported the notion that improper protein levels can negatively affect prawns' overall health and stress resilience.

## 5. Conclusion

Inappropriate and excessive protein levels negatively affected the growth performance, feed utilization efficiency, digestive enzyme activity, and AOC of *M. rosenbergii*. Obvious hepatopancreatic injury was also observed in prawns with excess dietary protein. From the perspective of growth and health, the appropriate dietary protein level for *M. rosenbergii* is suggested to be 42.5%–45%, with a culture density of 70 prawns/m^3^. As a matter of fact, this study is a proposal for dietary protein supply for prawns based on the industrial aquaculture practice with a high-density culture model. The findings will not only aid in the formulation of more efficient and cost-effective feeds but also contribute to the long-term sustainability of *M. rosenbergii* aquaculture by minimizing environmental impacts, optimizing feed usage, and improving production efficiency. Our laboratory is currently evaluating the impacts of this culture model on pond water loading and other aspects, aiming at a combination of both economic and ecological benefits, to achieve the sustainable development of the industry.

## Figures and Tables

**Table 1:** Ingredients and composition of experimental diets (% dry matter).

Items	Groups				
	CP40	CP42.5	CP45	CP47.5	CP50
Soybean meal (45.8% crude protein)	19.00	19.00	20.00	21.00	22.00
Fish meal-imported Japanese grade (70.4% crude protein)	17.00	18.00	19.00	19.50	21.00
Fish meal-domestic semi-skimmed (68.8% crude protein)	11.00	12.00	13.00	14.00	15.00
Shrimp meal (61.2% crude protein)	7.00	8.00	9.00	10.00	11.00
Peanut meal (53.3% crude protein)	5.00	5.00	5.00	5.00	5.00
Poultry byproduct meal (65.8% crude protein)	1.50	2.00	2.50	3.00	3.50
Wheat flour (13.5% crude protein)	19.00	19.00	19.00	18.10	13.30
Soybean oil	0.75	0.70	0.45	0.30	0.10
Fish oil	0.75	0.50	0.45	0.30	0.10
Shrimp paste	2.00	2.00	2.00	2.00	2.00
Squid paste	2.00	2.00	2.00	2.00	2.00
Soybean lecithin	2.50	2.50	2.50	2.50	2.50
Ca(H_2_PO_4_)_2_	1.50	1.50	1.50	1.50	1.50
Choline chloride -60%	0.25	0.25	0.25	0.25	0.25
^a^Vitamin mixture	0.20	0.20	0.20	0.20	0.20
^b^Mineral mixture	0.20	0.20	0.20	0.20	0.20
Vitamin C–phosphate	0.10	0.10	0.10	0.10	0.10
Organic acid	0.05	0.05	0.05	0.05	0.05
Bentonite	10.20	7.00	2.80	0.00	0.00
Guar gum	0.00	0.00	0.00	0.00	0.20
Total	100.00	100.00	100.00	100.00	100.00
*Proximate composition* (*% dry matter*)					
Moisture	10.76	10.79	10.79	10.46	10.19
Crude protein	40.44	42.13	45.06	47.63	49.94
Crude lipid	8.25	8.20	7.92	8.02	7.96
Ash content	18.79	14.91	9.82	7.84	7.26

^a^Vitamin mixture (IU or g/kg diet): biotin, 0.060 g; folic acid, 0.15 g; nicotinic acid, 3.5 g; pyridoxine hydrochloride, 0.6 g; riboflavin, 0.7 g; thiamin, 0.5 g; vitamin A, 450,000 IU; vitamin B_12_, 0.002 g; vitamin D_3_, 150,000 IU; vitamin E, 5 g; vitamin K_3_, 0.5 g.

^b^Mineral mixture (g/kg diet): Ca(H_2_PO_4_)_2_, 10 g; CoCl_2_ · 6H_2_O, 4.8 mg; CuSO_4_ · 5H_2_O, 40 mg; FeSO_4_ · H_2_O, 155 mg; KCl, 4.5 g; KI, 11.7 mg; MgSO_4_ · 7H_2_O, 2.4 g; MnSO_4_ · H_2_O, 30 mg; Na_2_SeO_3_, 2.4 mg; NaCl, 2.1 g; ZnSO_4_ · H_2_O, 80 mg.

**Table 2:** Primers used for the quantitative real-time PCR analysis of target genes in *M. rosenbergii*.

Gene	Position	Primer sequence	Length
*β-actin*	Forward (5′–3′)	GTGGTCGTGAAGGTGTAGCC	20
	Reverse (5′ –3′)	GAGGGGTATGCACTTCCTCA	20
*hsp60*	Forward (5′ –3′)	TCTCGGCAAGTAACCACTCC	20
	Reverse (5′ –3′)	CCATCACGACCAACCTTCTC	20
*hsp70*	Forward (5′ –3′)	CCTCTGCCCAAGCAAGTATT	20
	Reverse (5′ –3′)	TACCACGGAACAAGTCACCA	20
*hsp90*	Forward (5′ –3′)	ATGAACGACGAGACCGAGAC	20
	Reverse (5′ –3′)	GGCATCTGAGGAGTTGGAGA	20
*caspase3*	Forward (5′ –3′)	AGGGGATAAAACCGATGGAG	20
	Reverse (5′ –3′)	CCCACATGACGAGCATATCA	20
*bcl-2*	Forward (5′ –3′)	CGCCACAGTAGGAGAGAAGG	20
	Reverse (5′ –3′)	TGAAAACGGCAATGGACATA	20
*caspase2*	Forward (5′ –3′)	CAGAGTGGGTTATGGGGCTA	20
	Reverse (5′ –3′)	ATCGGTTCTTGCATCCTGAC	20
*nf-κb*	Forward (5′ –3′)	GTGGCTCACTTACGACTC	18
	Reverse (5′ –3′)	AAGGTCCATACTCTTTGC	18

Abbreviations: *bcl-2*, b-cell lymphoma-2*; hsp60*, heat shock protein 60*; hsp70*, heat shock protein 70*; hsp90*, heat shock protein 90*; nf-κb*, nuclear factor kappa-B.

**Table 3:** Effects of different dietary protein levels on the growth performance, feed utilization, and survival rate of *M. rosenbergii*.

Items	Groups					SEM	*p*-Value		
	CP40	CP42.5	CP45	CP47.5	CP50		ANOVA	Linear	Quadratic
*W*_i_ (g)	0.76	0.76	0.76	0.76	0.76	0.003	0.555	0.897	0.315
*W*_f_ (g)	11.90^a^	12.76^b^	13.24^b^	11.69^a^	12.06^a^	0.158	<0.001	0.296	0.002
WGR (%)	1464.17^a^	1582.31^c^	1640.91^b^	1441.21^a^	1484.85^a^	21.001	<0.001	<0.001	0.002
SGR (%)	3.62^a^	3.71^b^	3.76^b^	3.60^a^	3.64^a^	0.017	<0.001	<0.001	0.002
FCR	2.65^b^	2.43^a^	2.35^a^	2.65^b^	2.64^b^	0.040	0.013	0.008	0.048
HSI (%)	2.92^ab^	3.10^abc^	3.46^c^	2.87^a^	3.40^bc^	0.086	0.047	0.164	0.646
CF (%)	1.19^b^	1.13^a^	1.21^b^	1.14^a^	1.20^b^	0.010	0.008	0.612	0.104
FI (g)	0.38	0.38	0.38	0.38	0.38	0.002	0.748	0.390	0.331
SR (%)	77.78	78.33	77.78	77.78	76.67	0.341	0.681	0.775	0.884

*Note:* Values in the same column with different superscript letters are significantly different (*p* < 0.05). CP42.5, 42.5% protein; CP45, 45% protein; CP47.5, 47.5% protein; CP50, 50% protein; CP40, 40% protein; *W*_i_, average initial weight; *W*_f_, average final weight.

Abbreviations: CF, condition factor; FCR, feed conversion ratio; FI, feed intake; HSI, hepatosomatic index; SEM, standard error of the mean; SGR, specific growth rate; SR, survival rate; WGR, weight gain rate.

**Table 4:** Effects of different protein levels on biochemical indicators in the hepatopancreas of *M. rosenbergii*.

Items	Diets					SEM	*p*-Value		
	CP40	CP42.5	CP45	CP47.5	CP50		ANOVA	Linear	Quadratic
T-AOC (mmol/g prot)	0.25^a^	0.30^b^	0.37^c^	0.35^c^	0.34^c^	0.013	<0.001	<0.001	<0.001
CAT (U/mg prot)	11.90^a^	12.76^c^	13.24^d^	11.69^b^	12.06^a^	0.012	<0.001	0.781	<0.001
T-SOD (U/mg prot)	47.92^a^	49.00^a^	74.74^c^	63.52^b^	47.87^a^	3.091	<0.001	0.151	<0.001
PC (nmol/mg prot)	6.86^c^	4.57^b^	2.16^a^	4.81^b^	6.48^c^	0.396	<0.001	0.522	<0.001
MDA (nmol/mg prot)	1.26^bc^	1.03^a^	1.15^ab^	2.04^d^	1.35^c^	0.067	<0.001	<0.001	0.345
GST (U/mg prot)	53.75^a^	56.19^a^	75.11^c^	74.11^c^	65.02^b^	2.292	<0.001	<0.001	<0.001

*Note*: Values in the same column with different superscript letters are significantly different (*p* < 0.05). CP42.5, 42.5% protein; CP45, 45% protein; CP47.5, 47.5% protein; CP50, 50% protein; CP40, 40% protein;

Abbreviations: CAT, catalase; GST, glutathione S-transferase; MDA, malondialdehyde; PC, protein carbonyl; SEM, standard error of the mean; T-AOC, total antioxidant capacity; T-SOD, total superoxide dismutase.

**Table 5:** Effects of different protein levels on hematologic immune indicators of *M. rosenbergii*.

Items	Diets					SEM	*p*-Value		
	CP40	CP42.5	CP45	CP47.5	CP50		ANOVA	Linear	Quadratic
NO (umol/L)	89.39^c^	103.81^d^	100.01^d^	82.75^b^	70.37^a^	2.883	<0.001	<0.001	<0.001
TP (g/L)	183.51^ab^	220.92^c^	203.82^bc^	162.31^a^	169.38^ab^	7.101	0.018	0.036	0.066
ALT (U/L)	24.15^d^	6.57^a^	10.34^b^	15.00^c^	17.08^c^	1.557	<0.001	0.019	<0.001
AST (U/L)	54.96^c^	49.26^b^	45.51^ab^	42.10^a^	47.14^ab^	1.217	0.005	<0.001	0.005
ALB (g/L)	52.19^c^	53.59^c^	52.58^c^	45.11^a^	49.52^b^	0.825	<0.001	<0.001	0.881

*Note:* Values in the same column with different superscript letters are significantly different (*p* < 0.05). CP40, 40% protein; CP42.5, 42.5% protein; CP45, 45% protein; CP47.5, 47.5% protein; CP50, 50% protein.

Abbreviations: ALB, albumin; ALT, alanine aminotransferase; AST, aspartate aminotransferase; NO, nitric oxide; SEM, standard error of the mean; TP, total protein.

## Data Availability

The data that support the findings of this study are available from the corresponding author upon reasonable request.
